# Age, absolute CD4 count, and CD4 percentage in relation to HPV infection and the stage of cervical disease in HIV-1-positive women

**DOI:** 10.1097/MD.0000000000019273

**Published:** 2020-02-28

**Authors:** Ramadhani Chambuso, Raj Ramesar, Evelyn Kaambo, Alltalents T. Murahwa, Mohammed O.E. Abdallah, Michelle De Sousa, Lynette Denny, Anna-Lise Williamson, Clive M. Gray

**Affiliations:** a MRC Unit for Genomic and Precision Medicine, Division of Human Genetics, Department of Pathology; b Department of Gynaecology, Morogoro Regional Referral Hospital, Morogoro, Tanzania; c Division of Human Genetics; d Division of Medical Virology, Department of Pathology, Faculty of Health Sciences; e Department of Biochemistry and Medical Microbiology, University of Namibia School of Medicine, Windhoek, Namibia; f Department of Obstetrics and Gynaecology, Victoria Wynberg Hospital, Cape Town; g South African Medical Research Council, Clinical Gynaecological Cancer Research Centre; h Department of Obstetrics and Gynaecology; i Division of Immunology, Institute of Infectious Disease and Molecular Medicine and Department of Pathology, University of Cape Town; j National Health Laboratory Service, Groote Schuur Hospital, Cape Town, South Africa.

**Keywords:** age and cervical cancer, CD4 count and CD4 percentage disconnect, cervical tumor biopsies, HIV-1/HPV co-infection, HPV genotypes

## Abstract

A subgroup of women who are co-infected with human immunodeficiency virus type 1 (HIV-1) and human papillomavirus (HPV), progress rapidly to cervical disease. We characterized HPV genotypes within cervical tumor biopsies, assessed the relationships of cervical disease stage with age, HIV-1 status, absolute CD4 count, and CD4 percentage, and identified the predictive power of these variables for cervical disease stage in a cohort of South African women.

We recruited 181 women who were histologically diagnosed with cervical disease; 87 were HIV-1-positive and 94 were HIV-1-seronegative. Colposcopy-directed tumor biopsies were confirmed by histology and used for genomic DNA extraction. The Roche Linear Array HPV genotyping test was used for HPV genotyping. Peripheral whole blood was used for HIV-1 rapid testing. Fully automated FC500MPL/CellMek with PanLeucogate (PLG) was used to determine absolute CD4 count, CD4 percentage, and CD45 count. Chi-squared test, a logistic regression model, parametric Pearson correlation, and ROC curves were used for statistical analyses. We used the Benjamini-Horchberg test to control for false discovery rate (FDR, q-value). All tests were significant when both *P* and q were <.05.

Age was a significant predictor for invasive cervical cancer (ICC) in both HIV-1-seronegative (*P* < .0001, q < 0.0001) and HIV-1-positive women (*P* = .0003, q = 0.0003). Sixty eight percent (59/87) of HIV-1-positive women with different stages of cervical disease presented with a CD4 percentage equal or less than 28%, and a median absolute CD4 count of 400 cells/μl (IQR 300–500 cells/μl). Of the HIV-1-positive women, 75% (30/40) with ICC, possessed ≤28% CD4 cells vs 25% (10/40) who possessed >28% CD4 cells (both *P* < .001, q < 0.001). Furthermore, 70% (28/40) of women with ICC possessed CD4 count >350 compared to 30% (12/40) who possessed CD4 count ≤ 350 (both *P* < .001, q < 0.001).

Age is an independent predictor for ICC. In turn, development of ICC in HIV-1-positive women is independent of the host CD4 cells and associates with low CD4 percentage regardless of absolute CD4 count that falls within the normal range. Thus, using CD4 percentage may add a better prognostic indicator of cervical disease stage than absolute CD4 count alone.

## Abstract 2

Supplemental Digital Content is available in the text

## 1 Introduction

Cervical cancer is the most common cause of cancer-related morbidity and mortality,^[1,2]^ and the most common AIDS-related cancer in women in sub-Saharan Africa.^[3,4]^ Women who are infected with human immunodeficiency virus type 1 (HIV-1), are more likely to have a higher prevalence of genital oncogenic human papillomavirus (HPV) infection than women who are not infected with HIV.^[5,6]^ Persistent infection of HPV can lead to the development of cervical precancerous lesions.^[7]^ These precancerous lesions can progress to invasive cervical cancer (ICC) if not treated or without an effective immune response to clear the persistent oncogenic HPV infection.^[8]^

HPV-infection screening is conducted to identify HPV genotypes causing the lesion and proof of viral replication in the cervical lesion.^[9,10]^ Partial genotyping is recommended when HPV screening is used.^[11]^ In HIV-1-positive women with cervical disease, multiple infections with different HPV genotypes are common.^[6]^ Additionally, genotype-specific HPV burden from the primary cervical lesion may determine the progression of pre-invasive to invasive cancer.^[6,12,13]^ However, there is limited data on HPV genotypes associated with cervical abnormalities in HIV-1-positive women drawn from the tumor itself in correlation with immune cell markers, such as absolute CD4 count and CD4 percentage.^[9,14–16]^

Within the tumor, extrachromosomal HPV viral genomes often become integrated into the host genome. This integration event is thought to drive oncogenesis by dysregulating expression of the *E6* and *E7* viral oncogenes, leading to inactivation of critical cell-cycle checkpoints and increased genomic instability in the host.^[17,18]^ However, many previous studies used only cytology results synonymously with cervical premalignant changes and none of them correlated with absolute CD4 count and CD4 percentage, concurrently.^[19–21]^ Because cytology is only a screening test, it is important that the oncogenic HPV genotypes be confirmed inside the tumor itself.^[15]^ Lack of characterization of age, absolute CD4 count and CD4 percentage concurrently in women with cervical disease, makes the association of diagnostic clinical immune parameters in women with cervical disease unclear, particularly in HIV-1 infected women in South Africa, where HIV-1 prevalence and incidence in women are among the highest in the world.^[22]^

The aim of this study was to characterize HPV genotypes within cervical tumor biopsies, to assess the relationships of cervical disease stage with age, HIV-1 infection status, absolute CD4 count, and CD4 percentage, and to identify the predictive power of these variables for cervical disease stage in a cohort of South African women.

## 2 Methods

### 2.1 Research ethics

All procedures performed in this study involving human participants were in accordance with the ethical standards of the institutional and/or national research committee and with the 1964 Helsinki declaration and its later amendments or comparable ethical standards. Ethical approval was granted from the Human Research Ethics Committee (HREC) of the University of Cape Town (HREC 903/2015). All methods were performed in accordance with the relevant guidelines and regulations of the Departments of gynecology of all respective hospitals and Provincial Department of Health of the Western Cape Province and the South African National Health Laboratory Service (NHLS). Subjects were recruited with informed consent: Written and signed consent forms in the language of the subjects choice were obtained in the presence of a witness. This was after detailed discussion with patients regarding the aims and nature of the study. A trained Registered Professional Nurse who was fluent in the relevant languages explained the details of the study and answered questions from the patients before their consent was requested.

### 2.2 Study design, subjects, HIV testing, and CD4-T-lymphocyte enumeration

As part of a large ongoing hospital-based project, this cross-sectional study recruited 181 women with histologically confirmed cervical disease from the Groote Schuur Hospital, Somerset Hospital and Victoria Wynberg Hospital in Cape Town, in the Western Cape Province of South Africa. All participants were above 18 years of age, and the recruitment process was conducted from June 2016 to March 2017. All women were referred from peripheral health centers to these 3 hospitals after cervical screening with abnormal Pap smear or suspicions of cervical malignancy after gynecological examination. Patients were recruited from the outpatient gynecological cancer assessment clinics, colposcopy clinics and the gynecological emergency rooms. Inclusion criteria were newly diagnosed patients with cervical disease and ability to consent. Using colposcopy inspection, Gynecologists collected punch biopsies of abnormal cervical lesions. The biopsied tissues tissues were stored in transport medium and sent to the Anatomical Pathology laboratory for histopathological analyses. The staging summary of cervical disease presented in this study was done as recommended by the revised FIGO staging guidelines.^[23]^

According to the South African HIV-testing algorithm, peripheral whole blood (4 ml) was collected in EDTA tubes (BD Vacutainer, South Africa). Approximately 20 μl of the collected peripheral whole blood was used for rapid HIV-1 antibody testing (Determine, Alere, Inc., Johannesburg, South Africa).^[24]^ All HIV-1-positive women were on antiretroviral therapy (ART) and the absolute CD4 count, CD4 percentage and CD45 white cell count were performed automatically using fully automated FC500MPL/CellMek system with a PanLeucogate (PLG) (modified gating strategy) platform as described elsewhere by Coetzee et al.^[25]^ Generally, a CD4 percentage of 14% to 28% corresponds to an absolute CD4 count between 200 and 500 cells/μl.^[26]^ The cut-off for CD4 percent depends on the region and inter-assay or inter-laboratory variability.^[27–29]^ For example Guiguet et al, used a cut-off of below or equal to 20% and above 20% in their study population in France.^[30]^ However, in South Africa, the laboratory CD4% reports show in a cut-off as below or equal to 28% and above 28%.

### 2.3 HPV DNA detection and typing

Genomic DNA was extracted from histologically-confirmed cervical tumor specimens by using reagents in the Qiagen QIAamp DNA Mini purification kit (Qiagen, Johannesburg, South Africa) according to the manufacturers protocol. The extracted DNA was quantified using a Nanodrop Spectrophotometer (Thermo Fisher Scientific, Johannesburg, South Africa). Due to the high concentration of genomic DNA from the tissue biopsies, the DNA was diluted using nuclease free water (Thermo Fisher Scientific, Johannesburg, South Africa) to reach a recommended final concentration of 0.2 ng/μl. The PCR-based Roche Linear Array HPV genotyping test (Roche Molecular Systems, Pleasanton, CA, USA) which identifies and distinguishes about 37 different HPV genotypes (HPV-6, -11, -16, -18, -26, -31, -33, -35, -39, -40, -42, -45, -51, -52, -53, -54, -55, -56, -58, -59, -61, -62, -64, -66, -67, -68, -69, -70, -71, -72, -73, -81, -82, -83, -84, -89 (HPV-CP6108) and -IS39) was used for typing HPV according to the manufacturers instructions.

### 2.4 Statistical analysis

All statistical analyses were performed as recommended in these previous studies.^[31,32]^ We used Chi-Squared test with descriptive statistics to summarize the simple statistics in different stages of cervical disease according to different clinical predictor variables such as age, HPV and HIV-1 infections, age at sexual debut, parity, and tobacco use. For HIV-1-positive women, in order to study immune predictor variables and age of patients, concurrently, we used a multivariable logistic regression model with cervical disease as a dependent categorical variable and mild cervical intraepithelial neoplasia (CIN 1) as a reference category to show the relationship of the stage of cervical disease with age, absolute CD4 count, CD4 percentage, and CD45 count for each stage of cervical disease. The correlation between absolute CD4 count and CD4 percentage was performed using parametric Pearson correlation analyses. We used receiver operating characteristic (ROC) curves to assess sensitivity and specificity by calculating the area under the curves (AUC) to predict disease stage outcome using the GraphPad Prism 8 software (www.graphpadPrism.com). Normal distribution 2 proportion z-test was used to test for statistically significant differences between 2 proportions within the same categorical group. All *P* values were corrected for False Discovery Rate (FDR) by the Benjamini-Hochberg method and the adjusted *P* values (q-values) were reported. All odds ratios (ORs), 95% confidence intervals (95% CIs) and the *P* values calculated for multiple comparisons were 2-tailed, and considered significant if both *P* and q were <.05.

## 3 Results

### 3.1 Characteristics of the study cohort

The study cohort was comprised of 181 women histologically diagnosed with cervical disease; 87 HIV-1-positive and 94 HIV-1-seronegative. The clinical predictor variables in our study population were: age in years, HPV infection, HIV infection, parity (number of children), and the tobacco use, consistent with previous published reports.^[33,34]^ Of this study cohort: about 18.8% were documented with the age of sexual debut below 16 years, while 85.5% were positive for HPV infection by DNA test and 67.8% who were diagnosed with ICC were HIV-1-positive. There were significant associations between age above 40 (*P* = .021, q = 0.063), HIV-1-positive status (both p and q < 0.001), and having more than four (4) children (both p and q < 0.001), with ICC (Table 1).

**Table 1:**
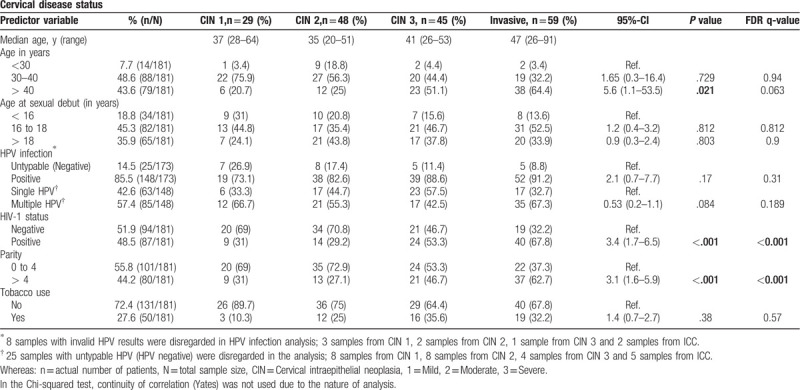
Demographics and the range of variables including clinical predictors measured, and the stages of cervical disease in the subjects of this study.

### 3.2 Relationships between specific HPV genotypes, HIV status, and tumor stages within different age groups

**Figure 1:**
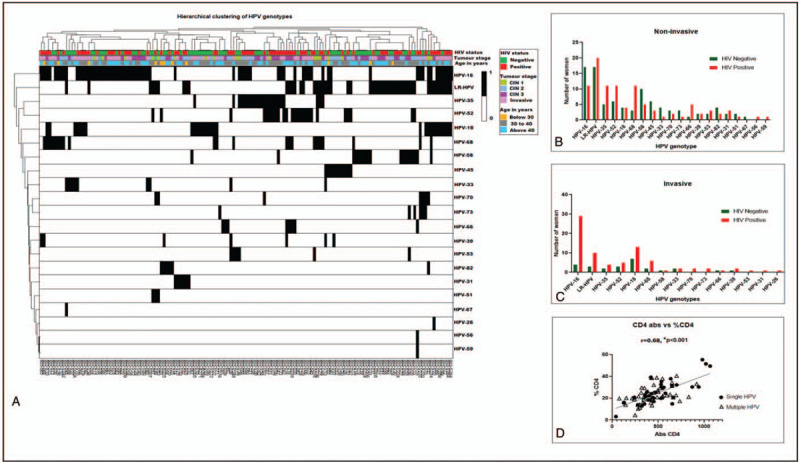
Illustration of HIV-1 status, HPV genotypes, and age groups at different stages of cervical disease. (**A**) Unsupervised hierarchical clustering showing HPV genotype distribution according to HIV-1 status (green = HIV-1-soronegative: red = HIV-1-positive), tumor stage (CIN 1-3 and invasive, and age in years, <30; 30–40; >40). The X-axis represents individual biopsy samples and the Y-axis shows HPV genotypes. The presence of a specific HPV genotype is shown as solid black bars (1) and no color (0) represents the absence of HPV infection (specific genotype). The color bars above the map indicate the HIV-1 status, tumor stage and age groups in years. (B &C) Histograms showing the number of women with specific HPV genotypes with precancerous lesions and with ICC, respectively. (D) Pearson correlation between absolute CD4 count and CD4 percentage in women with single HPV genotype (solid circles) and multiple HPV genotypes (open triangles). -Some women may be counted more than once due to multiple HPV infections. Where; CIN is cervical intraepithelial neoplasia, 1 = mild, 2 = moderate, 3 = severe. Color blocks do not represent the actual number of patients. Hr-HPV = High risk HPV: HPV-16,-18,-31,-33,-35,-39,-45,-51,-52,-56,-59. PHr-HPV = Probable high risk HPV: HPV-26,-53,-66,-67,-68,-70,-73 and -82. Lr-HPV = Low risk HPV: HPV-6,-11,-40,-42,-54,-55,-61,-62,-64,-69,-71,-72,-81,-83 (HPV-CP6108) and –IS39.

Age, HPV genotypes, and HIV-1 status at different stages of cervical disease were investigated in the research cohort. As anticipated, HPV-16 was the most frequently genotype either as a single infection or in mixed infections with other HPV genotypes. Furthermore, HPV-16 genotype was present at a higher frequency in the ICC biopsies from HIV-1-positive patients (72.5%, 29/40) than in the ICC biopsies from HIV-1-seronegative women (21.1%, 4/19). There was a moderate correlation between absolute CD4 count and CD4 percentage, irrespective of single or multiple HPV infections (r = 0.68, *P* < .001). Collectively, the results show the presence of mixed HPV infections in both HIV-1-positive and HIV-1-seronegative women (Fig. 1A–D).

### 3.3 Comparison between CD4 status, HPV infection, and cervical disease stage in HIV-1-positive patients.

**Figure 2:**
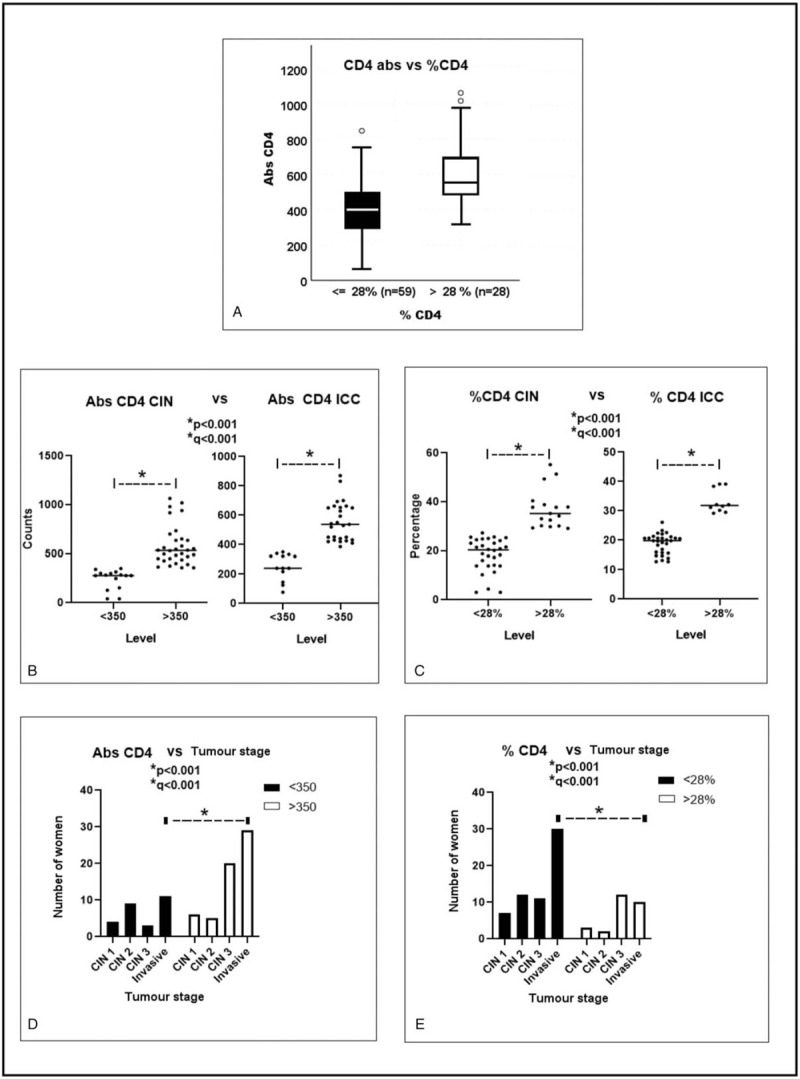
Relationships between absolute CD4 count (Abs CD4), CD4 percentage (% CD4), HPV single or multiple infections, number of women and cervical disease stages in HIV-1-positive women. (A) Box and whisker plots showing the relationship between % CD4 percentage and Abs CD4 with number of patients and median CD4 count. (B) Scatter plots showing comparisons of levels of Abs CD4 between CIN and ICC (C) Scatter plots showing comparisons of % CD4 CIN and ICC (D) Histograms showing number of women and comparisons of tumor stages and Abs CD4 within cervical disease groups (F) Histograms showing number of women and comparisons between tumour stage and % CD4 within cervical disease groups. Where; R^2^ = is the proportion of the variance (coefficient of determination), *P* = statistical power and q = Benjamini-Horchberg false discovery rate.

Previous studies have shown that in some women, the stage of clinical immunosuppression during HIV infection does not associate with stage of cervical disease or ICC.^[35]^ Equally, high CD4 count and antiretroviral therapy do not appear to prevent HPV infection and cervical disease development.^[6,35]^ Consequently, the relationship between absolute CD4 count, CD4 percentage, HPV infection and stage of cervical disease in the HIV-1-positive women group was examined in the group of HIV-1-positive women in this study. Approximately, 68% (59/87) of HIV-1-positive women with different stages of cervical disease presented with a CD4 percentage below or equal to 28% and a median absolute CD4 count of 400 cells/μl (Inter quartile range, IQR, 300–500 cells/μl (Fig. 2A). When we examined the association between absolute CD4 count vs CD4 percentage, there were significant moderate to strong correlations in all CIN (overall r = 0.68, *P* < .001; for CIN r = 0.83, *P* < .001) and a weak correlation in ICC (r = .32, *P* = .043) (Supplementary Figs. 1A–C). Cervical disease was more prevalent in women with low CD4 percentage than in women with high CD4 percentage, regardless of absolute CD4 count (Fig. 2B and C). Moreover, there was a significant difference found in absolute CD4 count and tumor stage. Larger proportion of women with ICC (70%, 28/40) possessed CD4 count higher than 350 compared to 30% (12/40) who possessed CD4 count ≤350 (*P* < .001, q < 0.001, Fig. 2D). Furthermore 75% (30/40) of women with ICC, possessed a CD4 percentage ≤28 vs 25% (10/40) who possessed CD4 percentage >28% (*P* < .001, q < 0.001, Fig. 2E). Notably, high absolute CD4 count ≥350 cells/μl was not protective against CIN 3 and ICC despite significant differences in CD4 count (All *P* < .001, q < 0.001, Fig. 2D). Additionally, there was no significant difference between single and multiple HPV infections by immune cell status in both CIN and ICC (Supplementary Figs.2A–C).

### 3.4 Comparison between age of patients, CD4 status, and cervical disease in HIV-1-positive patients.

Aging and cancer are highly interconnected, ageing being a significant risk factor for cancer development.^[36]^ However, in cervical cancer, it has already been reported that HIV-1-positive women develop ICC earlier, and at a younger age compared to HIV-1-seronegative women.^[2,5,37]^ Since there is limited data on the impact of age and clinical immunological markers according to single or multiple HPV infections in cervical cancer development amongst HIV-1-positive women in Africa, we sought to investigate the relationship between stage of cervical disease, single or multiple HPV infections, age, absolute CD4 count and CD4 percentage in HIV-1-positive women by using parametric Pearson correlation test. Although there were no statistically significant correlations between age of patients and levels of CD4 count, as well as the age of patients and CD4 percentage, we observed an even distribution of HPV single or multiple (genotypes) infections, regardless of age, absolute CD4 count or CD4 percentage (Supplementary Figs. 3A and B).

To investigate the effects of multiple immune predictor variables and age of patients at different stages of cervical disease, we created a statistical model of risk by using multivariable logistic regression that adds age, absolute CD4 count, CD4 percentage, and CD45 white cell count as covariates to correct for multiple comparisons. Women older than 30 years had nearly 4^[4]^ times the odds of developing ICC (OR = 3.7, 95% CI = 1.5–13.1, *P* = .043), however, the q value was not significant (q = 0.172) (Table 2). Our results show that both CD4 count > 350 cells/μl and CD4 percentage >28% did not appear to likely prevent the risk of any cervical disease stage (all *P* > .05) (Table 2).

**Table 2:**
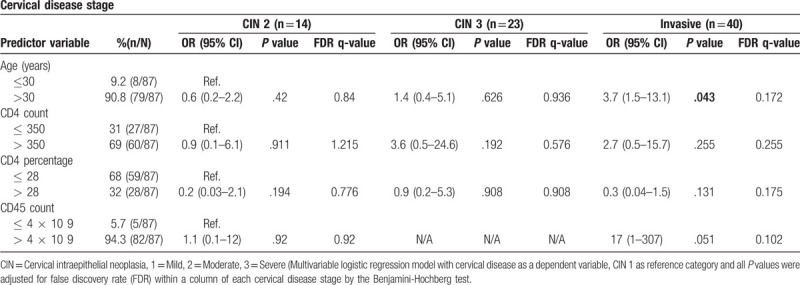
Cervical disease with different immune predictor variables and age of HIV-1-positive women.

**Figure 3:**
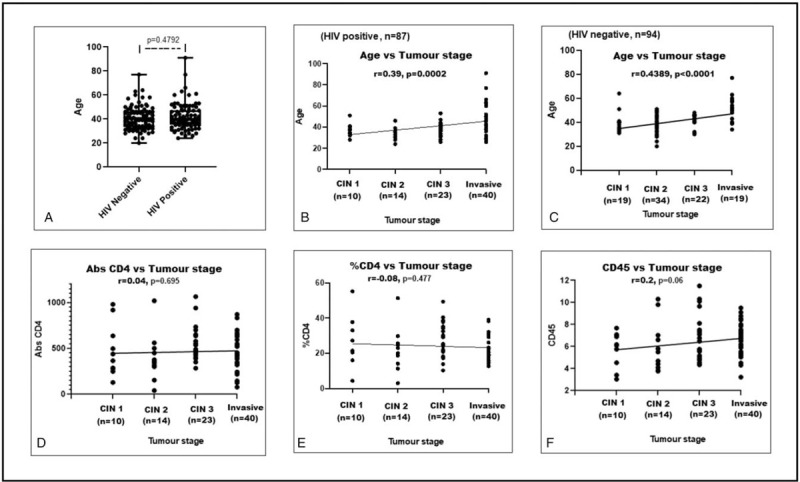
Relationships between age, absolute CD4 count (Abs CD4), CD4 percentage (%CD4), CD45 count (CD45), and tumor stages. (A) Box and whisker plots showing difference in age between HIV-1-positive and HIV-1-seronegative women with cervical disease. (B) Correlation between age and tumor stage in HIV-1-positive patients. (C) Correlation between age and tumor stage in HIV negative patients. (D, E, and F) Correlations between Abs CD4, %CD4, CD45, and tumor stage in HIV-1-positive patients.

We next wished to determine whether there was any relationship between stage of cervical disease, age, CD4 cell levels and HIV-1 status. We found that there was no significant age difference between HIV-1-positive and HIV-1-seronegative women (*P* = .4792, Fig. 3A). However, there were strong significant correlations between age and stage of cervical disease in both HIV-1-positive and HIV-1-seronegative women (*P* = .0002 and <.0001, respectively, Fig. 3B and C). There was no correlation between tumor stage and absolute CD4, CD4 percentage, or CD45 count (All *P* > .05, Fig. 3F).

**Figure 4:**
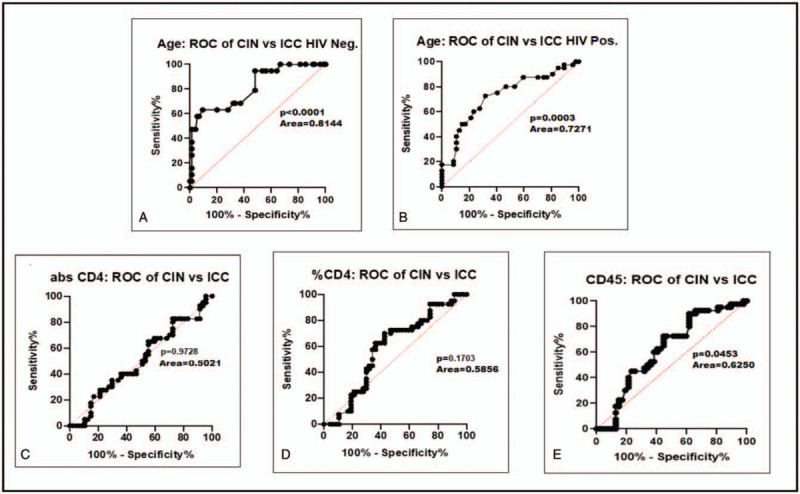
Receiver operating characteristic (ROC) curves between age, absolute CD4 count (Abs CD4), CD4 percentage (%CD4), CD45 count (CD45) in CIN vss ICC. (A) ROC curve of age between CIN vs ICC in HIV-1-seronegative women. (B) ROC of age between CIN vs ICC in HIV-1-positive women. (C) ROC curve of Abs CD4 between CIN vs ICC in HIV-1-positive women. (D) ROC curve of % CD4 between CIN vs ICC in HIV-1-positive women. (E) ROC curve of CD45 between CIN vs ICC in HIV-1-positive women.

Lastly, this study wished to determine whether age or the immune status of patients was predictive of cervical disease stage. Receiver operative characteristic (ROC) curves reflected that age was significantly predictive of whether a woman was likely to be diagnosed with any of the CIN stages, including ICC, and not absolute CD4 count, CD4 percentage, nor CD45 count. Thus, only age was an independent predictor of cervical disease for both HIV-1-positive and HIV-1-seronegative patients (*P* = .0003 and <.0001, respectively, Fig. 4 and supplementary Fig. 4).

## 4 Discussion

Our study shows the distribution of HPV genotypes from 181 histologically confirmed cervical tumor biopsies from HPV-unvaccinated women. We show that the distribution of HPV genotypes is related to the stage of cervical disease in HIV-1-positive women. We also relate the stage of cervical disease with age, HIV-1 infection, and immune status (as measured by absolute CD4 count, CD4 percentage, and CD45 count). There is a partial disconnect between absolute CD4 count and CD4 percentage in HIV-1-positive women with cervical disease, where more women with ICC were grouped with low CD4 percentage, irrespective of absolute CD4 count that falls within the normal range. This finding reveals some intriguing distinction in low CD4 percentage in HIV-1-positive women diagnosed with ICC that requires further investigation. We regard this as an important information because cervical disease progression differs significantly amongst HIV-1-positive women. It further suggests that other clinical immune cell markers apart from CD4 cells play a role in cervical disease progression.

This study is the first to report a partial disconnect between absolute CD4 count and CD4 percentage with cervical disease. There is also a novel association between low CD4 percentage and age (above 30 years) for HIV-1-positive women with ICC, where age was also significantly predictive of cervical disease stage. In fact, only age was an independent predictor for stage of cervical disease in both HIV-1-seronegative and HIV-1-positive women. These results suggest that, both age and CD4 percentage are important factors for cervical disease development, but only age is a strong predictor for ICC disease outcome. Previously, Denny et al,^[38]^ van Aardt et al,^[39]^ and Naucler et al,^[40]^ studied the distribution of HPV genotypes between HIV-1-positive and HIV-1-seronegative women, by using cervical tissue biopsies. However, they investigated only women with ICC and they did not consider the effects of age, CD4 count, CD4 percentage, and CD45 count concurrently, while the present study does.

In our study population, the significant positive linear correlation between absolute CD4 count and the CD4 percentage was not perfect (R = 0.69, R^2^ = 0.48) and this was interpreted as only a partial disconnect between absolute CD4 count and CD4 percentage. In clinical HIV studies, discordance between absolute CD4 count and the CD4 percentage has already been reported in HIV-infected patients depending on the study population.^[28,41–43]^ However, Anyimadu et al^[44]^ reported a discordance of 71.4% (20/28) in one of the sub-groups of HIV-infected patients. In our study, a discordance of about 68% (59/87) in HIV-1-positive women diagnosed pathologically with cervical disease was observed. The difference in correlation coefficients between absolute CD4 count and CD4 percentage with other variables may be due to varied immune cell enrichments in different patients. The discordance between absolute CD4 count and CD4 percentage suggests that other lymphocytes apart from CD4 cells may play role in the development cervical disease. This is because CD4 percentage shows the relationship of CD4 cells in consideration with white blood cell count and lymphocyte differential in the body.^[28,29]^ Apart from CD4 cells, other immune cells could play a role in the development of cervical disease include cytotoxic T cell (CD8^+^ T cells), B Lymphocytes, Natural killer cells, and other Natural killer T cells.^[45]^

An even distribution of single and multiple HPV infections, and cervical disease development, regardless of high CD4 count, was an observation similar to 2 previous reports.^[6,46]^ However, Denny et al^[47]^ found that having a CD4 count of more than 500/mm^3^ was protective against the development of cervical disease for 36 months, once the analysis was adjusted for age, sexual activity and HIV viral load. This discrepancy between our study and that of Denny et al,^[47]^ may be due to the fact that we did not consider patient follow up and HIV viral load in our study. However, de Jong et al,^[48]^ suggested that, there is an absence of functional HPV-16 specific CD4+ T-cell immune response in some women. This may justify and explain our findings of ICC despite competent CD4 count observed in some women. Since there is no level of absolute CD4 count that puts HIV-1-positive women at lower risk for persistent infection with oncogenic HPV and cervical disease development, we suggest that HIV-1-positive women may be ideal for individualized HPV vaccination of adult HIV-positive women, a similar suggestion as proposed by Dlamini et al.^[49]^

In HIV-1-positive women, HPV-16 genotype presented more in ICC biopsies than in non-invasive cancer biopsies (Fig. 1 B and C). However, we did not have enough sample size to assess the statistical significant difference. Future studies should focus on large sample size for this type of association analysis. Similar findings of high frequency of HPV-16 genotype infection as a single infection or in mixed infections with other HPV genotypes in HIV-1-positive women with cervical disease have been reported previously in different parts of the world, namely: Brazil,^[14]^ Burkina Faso, and South Africa in 1 study with samples from the 2 countries,^[50]^ Cameroon,^[51]^ South Africa,^[9,38,52]^ and the USA.^[53,54]^ Three studies; 1 from Mozambique,^[40]^ 1 from 2 countries Kenya and South Africa,^[55]^ and 2 from South Africa,^[56]^ did not find the association of HIV infection and HPV-16 genotype infection. However, 2 studies; the 1 from Kenya and South Africa, and the 1 from South Africa, used cervical swabs or brushes to collect cells/tissues around the cervix for HPV testing. Inconsistency in frequency of HPV-16 genotype in HIV-1-positive women using tumor biopsies and cervical swabs between our study and other studies, could be due to sample size difference, different study populations and possibility of cross contamination with other genital HPV genotypes during biopsy processing or swab collection.

The strengths of our study include: the use of histologically-confirmed cervical biopsies from precancerous lesions and invasive cancer, showing HPV distribution from cervical tumors from both unvaccinated HIV-1-positive and HIV-1-seronegative women and timely enumeration of CD4 cells in HIV-1-positive women. The limitations may include: Being a cross-sectional study, hospital-based studies are likely to have some selection bias, limited sample size, possibilities of genital HPV contamination during biopsy collection and processing, and the lack of focus on detailed information on immune status with regard to HIV-1 and HPV viral loads, consideration on the duration of ART, no follow up of absolute CD4 count, and the time of acquisition of HIV-1 or HPV infection was needed to be taken into account. Also there was no confirmatory testing for presence of HIV RNA in the studied tumor biopsies.

## 5 Conclusions

Our study suggests the presence of a partial disconnect between absolute CD4 count and CD4 percentage in a subgroup of HIV-1-positive women histologically diagnosed with cervical disease. HPV infection and cervical disease stage are irrelevant to the host immune status but cervical disease was more prevalent in women with low CD4 percentage regardless of absolute CD4 count that falls within the normal range. However, only age is a significantly independent predictor for ICC in both HIV-1-positive and HIV-1-seronegative women. Additional results of CD4 percentage should be used concurrently with absolute CD4 count to monitor cervical disease in HIV-1-positive women. Comprehensive investigation of low CD4 percentage with regard to HIV-1 and HPV viral loads in women with cervical disease is warranted in order to determine if this relationship is causal.

## Acknowledgments

The authors would like to thank;

i) Professor Tim Quinlan, from the Health Economics and HIV/AIDS Research Division (HEARD) at the University of KwaZulu-Natal, South Africa for his assistance in discussing and reviewing early drafts of this article.
ii) Rafiekah Abrahams from the Haematology Laboratory, CD4+ unit at the National Health Laboratory Service, Groote Schuur Hospital, Cape Town, South Africa.

## Author contributions

**Conceptualization:** Ramadhani Salum Chambuso, Evelyn Kaambo, Alltalents T Murahwa, Anna-Lise Williamson, Clive M Gray.

**Data curation:** Ramadhani Salum Chambuso, Evelyn Kaambo, Alltalents T Murahwa, Michelle De Sousa, Anna-Lise Williamson, Clive M Gray.

**Formal analysis:** Ramadhani Salum Chambuso, Evelyn Kaambo, Alltalents T Murahwa, Mohammed O.E Abdallah, Anna-Lise Williamson, Clive M Gray.

**Funding acquisition:** Ramadhani Salum Chambuso, Anna-Lise Williamson, Clive M Gray.

**Investigation:** Ramadhani Salum Chambuso, Raj Ramesar, Evelyn Kaambo, Anna-Lise Williamson, Clive M Gray.

**Methodology:** Ramadhani Salum Chambuso, Anna-Lise Williamson, Clive M Gray.

**Project administration:** Ramadhani Salum Chambuso, Raj Ramesar, Evelyn Kaambo, Anna-Lise Williamson, Clive M Gray.

**Resources:** Ramadhani Salum Chambuso, Evelyn Kaambo, Michelle De Sousa, Lynette Denny, Anna-Lise Williamson, Clive M Gray.

**Software:** Ramadhani Salum Chambuso, Mohammed O.E Abdallah, Clive M Gray.

**Supervision:** Ramadhani Salum Chambuso, Raj Ramesar, Lynette Denny, Anna-Lise Williamson, Clive M Gray.

**Validation:** Ramadhani Salum Chambuso, Anna-Lise Williamson, Clive M Gray.

**Visualization:** Ramadhani Salum Chambuso, Anna-Lise Williamson, Clive M Gray.

**Writing – original draft:** Ramadhani Salum Chambuso, Anna-Lise Williamson, Clive M Gray.

**Writing – review & editing:** Ramadhani Salum Chambuso, Anna-Lise Williamson, Clive M Gray.

Ramadhani Salum Chambuso orcid: 0000-0001-8794-604X.

## Supplementary Material

Supplemental Digital Content

## Supplementary Material

Supplemental Digital Content

## Supplementary Material

Supplemental Digital Content

## Supplementary Material

Supplemental Digital Content
