# Supplementary material for: Age, absolute CD4 count, and CD4 percentage in relation to HPV infection and the stage of cervical disease in HIV-1-positive women
Source: Medicine (Baltimore). 2020 Feb 28;99(9):e19273. doi: 10.1097/MD.0000000000019273 (PMC7478573; doi:10.1097/MD.0000000000019273)

**Supplementary Figure 1**. Scatter plots showing the relationships between absolute CD4 count (Abs CD4), CD4 percentage (%CD4) and CD45 count (CD45) in CIN and ICC. (**A**) Overall correlations between abs CD4 versus % CD4 versus CD45 count in all disease stages. (**B**) Correlations between abs CD4, % CD4 and CD45 only in invasive cancer. (**C**) Correlations between abs CD4, % CD4 and CD45 only in CIN.


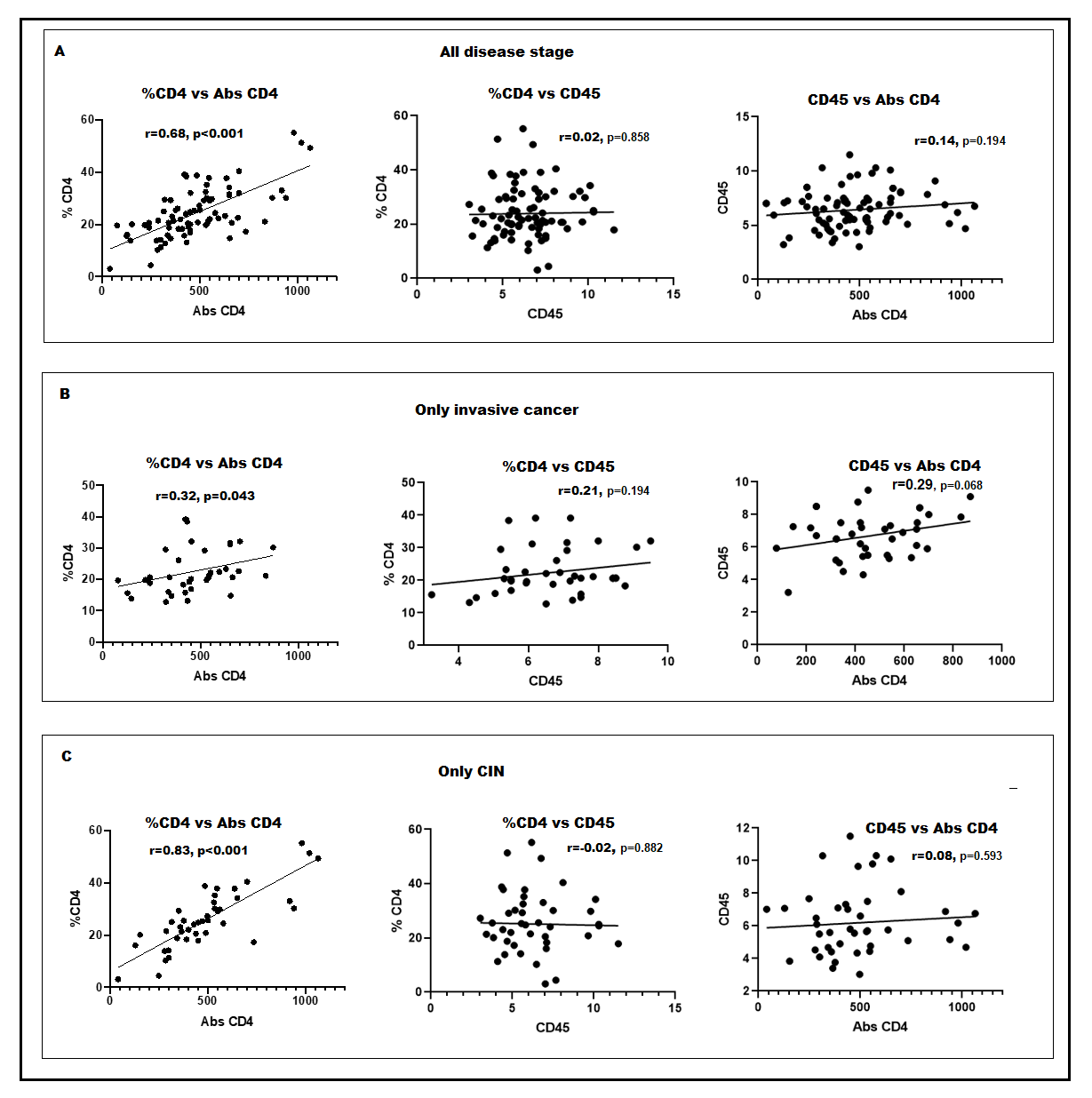

Supplement: Supplemental Digital Content [file medi-99-e19273-s001.docx]
