# Supplementary material for: Age, absolute CD4 count, and CD4 percentage in relation to HPV infection and the stage of cervical disease in HIV-1-positive women
Source: Medicine (Baltimore). 2020 Feb 28;99(9):e19273. doi: 10.1097/MD.0000000000019273 (PMC7478573; doi:10.1097/MD.0000000000019273)

**Supplementary figure 2**. Scatter plots showing the relationships between single and multiple HPV infections by absolute CD4 count (Abs CD4), CD4 percentage (%CD4) and CD45 count (CD45) in CIN and ICC. **(A)** Relationships between single and multiple HPV infections by abs CD4 in CIN and ICC **(B)** Relationships between single and multiple HPV infections by % CD4 in CIN and **(C)** Relationships between single and multiple HPV infections by CD45 in CIN and ICC.


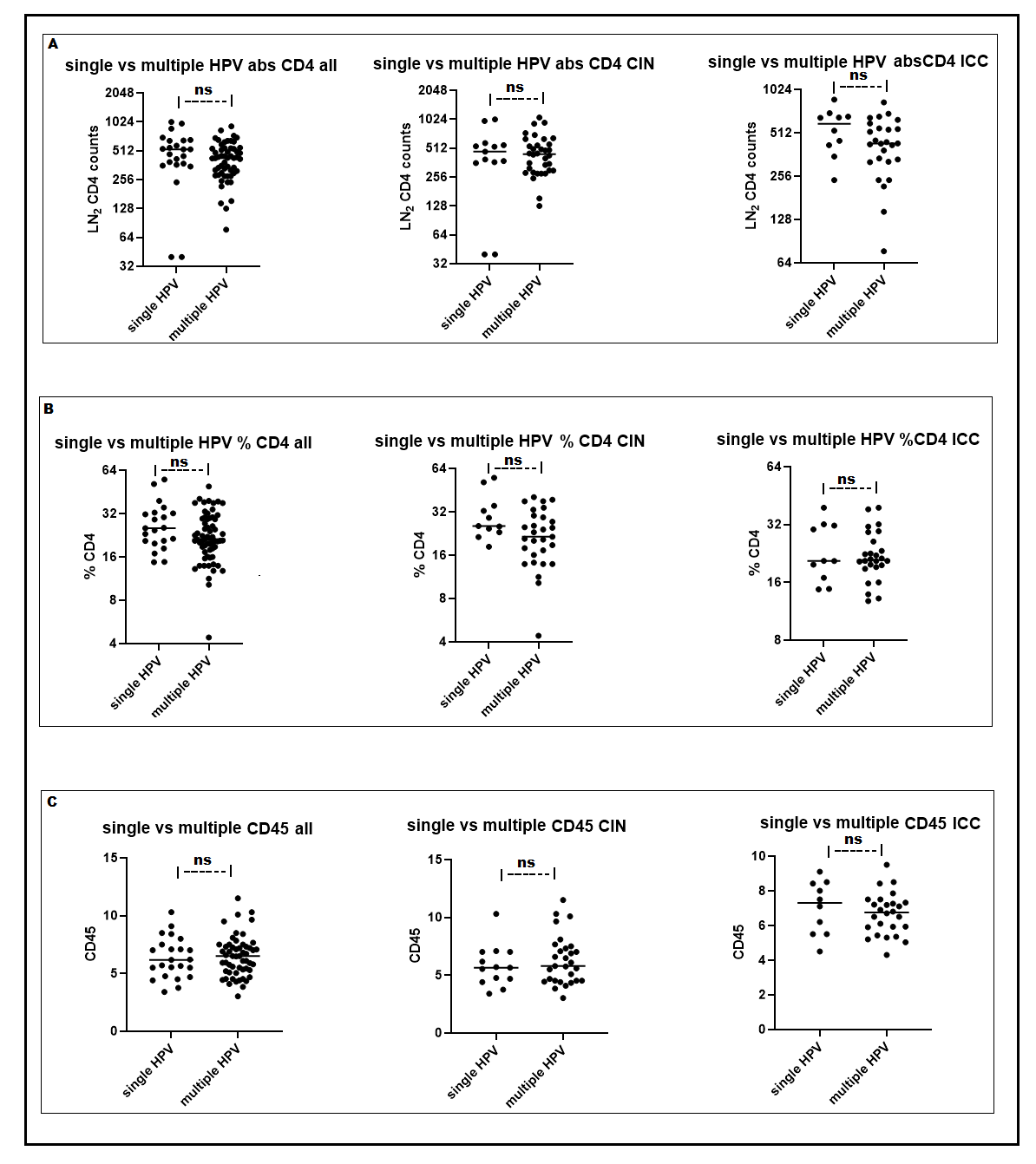

Supplement: Supplemental Digital Content [file medi-99-e19273-s002.docx]
