# Supplementary material for: Age, absolute CD4 count, and CD4 percentage in relation to HPV infection and the stage of cervical disease in HIV-1-positive women
Source: Medicine (Baltimore). 2020 Feb 28;99(9):e19273. doi: 10.1097/MD.0000000000019273 (PMC7478573; doi:10.1097/MD.0000000000019273)

**Supplementary Figure 3.** Relationships between age of patients and levels of immune cell markers. (A) Correlation between age and absolute CD4 count (Abs CD4). (B) Correlation between age and CD4 percentage (%CD4).


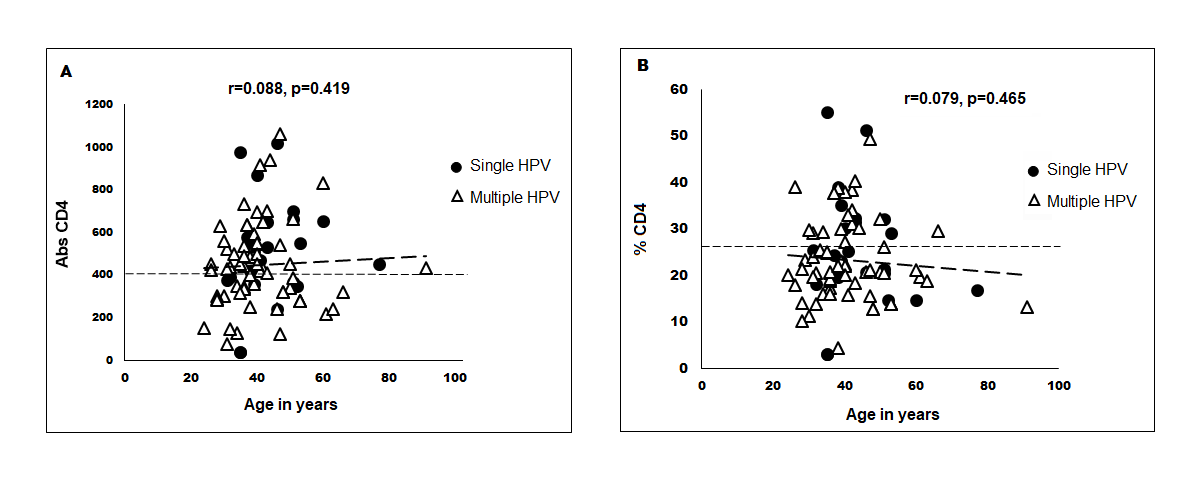

Supplement: Supplemental Digital Content [file medi-99-e19273-s003.docx]
