# Supplementary material for: Age, absolute CD4 count, and CD4 percentage in relation to HPV infection and the stage of cervical disease in HIV-1-positive women
Source: Medicine (Baltimore). 2020 Feb 28;99(9):e19273. doi: 10.1097/MD.0000000000019273 (PMC7478573; doi:10.1097/MD.0000000000019273)

**Supplementary Figure 4.** Receiver operating characteristic (ROC) curves between age, absolute CD4 count (Abs CD4), CD4 percentage (%CD4), CD45 count (CD45) in CIN 1 &2 versus CIN 3. (**A**) ROC curve of age between 1 &2 versus CIN 3 in HIV-1-seronegative women. (**B**) ROC of age between CIN 1 &2 versus CIN 3 in HIV-1-positive women. (**C**) ROC curve of Abs CD4 between CIN 1 &2 versus CIN 3 in HIV-1-positive women. (**D**) ROC curve of % CD4 between CIN 1 &2 versus CIN 3 in HIV-1-positive women. (**E**) ROC curve of CD45 between CIN 1 &2 versus CIN 3 in HIV-1-positive women.


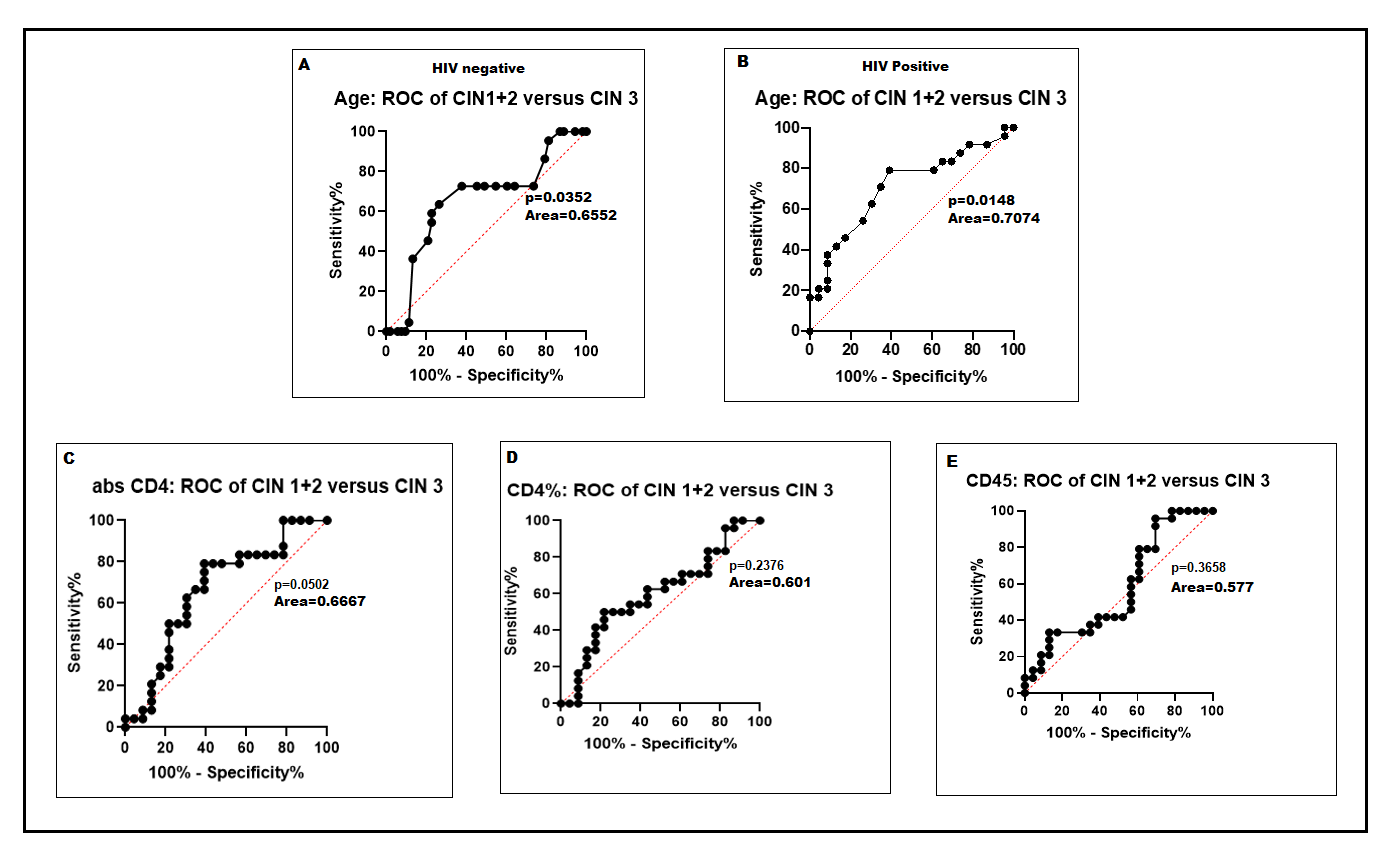

Supplement: Supplemental Digital Content [file medi-99-e19273-s004.docx]
